# Case report: Diagnosis of impaired consciousness in a cancer patient using immune checkpoint inhibitors

**DOI:** 10.3389/fimmu.2025.1458686

**Published:** 2025-01-31

**Authors:** Daniel Cathalifaud, Cristóbal Basáez, Tatiana Yáñez

**Affiliations:** ^1^ Escuela de Medicina, Pontificia Universidad Católica de Chile, Santiago, Chile; ^2^ Departamento de Medicina Interna, Hospital Clínico Red de Salud UC-Christus, Santiago, Chile; ^3^ Faculty of Medicine, Pontificia Universidad Católica de Chile, Santiago, Chile

**Keywords:** immune checkpoint inhibitor, delirium, psychoses, hyperthyroidism, thyrotoxicosis, mesothelioma

## Abstract

Immune checkpoint inhibitors (ICIs) are drugs that are being increasingly used in the field of oncology; due to their mechanism of action, they can present immune-related adverse effects (IRAEs), with various clinical manifestations, one of which is delirium. We present the case of a patient diagnosed with pleural mesothelioma that started combined palliative immunotherapy two months before admission. She was hospitalized for delirium with psychotic symptoms and a comprehensive neurological and etiological examination for this pathology was performed, revealing undetectable TSH levels, indicating the etiology of the condition as thyrotoxicosis in the context of autoimmune thyroiditis, secondary to treatment with ICIs. Symptomatic treatment with beta-blockers was initiated, leading to progressive improvement. This case brings awareness of impaired consciousness and neuropsychiatric symptoms as manifestation of IRAEs and the difficulty of their diagnosis: there may also be several other causes of impaired consciousness, so the characterization of delirium requires a multifaceted approach to determine the underlying cause, taking into account direct cancer-related complications and those stemming from the treatments received by this group of patients. Endocrinological immune-related adverse events (IRAEs), such as thyroid IRAEs, generally have a low lethality rate, do not necessarily require discontinuation of therapy, and are linked to a more favorable oncological prognosis. Conversely, neurological IRAEs, though rare, constitute a contraindication for further use of ICIs. This clinical case emphasizes the importance of the systematic study of consciousness impairment in cancer patients, and of considering multiple IRAEs that could lead to changes in oncological therapy when establishing possible etiologies.

## 1 Introduction

Immune checkpoint inhibitors (ICIs) are monoclonal antibodies that, as their name suggests, inhibit checkpoints in the immune system so as to restore immune response against tumor cells. Over the last decade, their use has expanded in the field of oncology because they can present equal or better results alone or in combination with conventional therapies, with lower rates of serious adverse effects and death (1). This class of medications includes antibodies targeting programmed death receptor 1 (PD-1), such as Nivolumab, and those directed against cytotoxic T-lymphocyte antigen 4 (CTLA-4), exemplified by Ipilimumab, among others (1). Given that ICIs disrupt immune system checkpoints and stimulate immune activation, they can induce the development of immune-related adverse effects (IRAEs). These adverse effects can occur in various systems, with several clinical manifestations (1).

Thus, multiple IRAEs can be related to impaired consciousness, including delirium (1). Delirium is a syndrome that poses challenges for clinicians, due to its multiple causes, and the need for a multifactorial approach (2). Notably, thyroid disorders can manifest with altered consciousness, particularly in the elderly (2, 3). Therefore, in the context of oncology patients receiving immune checkpoint inhibitors (ICIs), the differential diagnosis for delirium must encompass a broader spectrum of potential causes, including usual neurological ones (such as stroke, central nervous system infections, etc), systemic diseases (infections, myocardial infarctions, respiratory diseases, etc, just to name a few), and those derived of the underlying oncological processes, as well as the adverse effects of oncological interventions, including IRAEs.

The following section describes a case of delirium due to peripheral hyperthyroidism secondary to thyroiditis, deemed to be an IRAE caused by ICIs used for palliative treatment in a patient with mesothelioma. This case delves into the diagnostic process for cognitive impairment in individuals undergoing immune checkpoint inhibitor (ICI) therapy, shedding light on how immune-related adverse events (IRAEs) influence treatment strategies and clinical outcomes for these patients.

## 2 Case description

A 64-year-old female patient with a history of predominantly epithelioid pleural mesothelioma, treated with pemetrexed and cisplatin chemotherapy for 6 cycles between 2016-2017. At the 6th year of follow-up, she evolved with locoregional progression. Subsequently, she commenced palliative immunotherapy with Nivolumab at a dose of 180 mg (equivalent to 3 mg/kg) and Ipilimumab at 60 mg (corresponding to 1 mg/kg). Additional pertinent elements of her medical history comprised a history of inactive rheumatoid arthritis, fibromyalgia, depression, and rheumatic mitral valve disease. Her current medication regimen included methadone, morphine, celecoxib, pregabalin, esomeprazole, bisoprolol, sertraline, and clonazepam.

She was admitted to the hospital due to a 2-month history of progressive episodes of disorientation, inattention, frequent forgetfulness, confusion, sporadic nocturnal visual hallucinations, and occasional difficulties in naming objects, notable for the absence of evident aphasia but exhibiting fluctuating patterns throughout the day. The onset of symptoms coincided with the initiation of immunotherapy with nivolumab and ipilimumab. Additionally, she reported asthenia, adynamia, mild gait instability, heat intolerance, nocturnal sweating, palpitations, tremors, low mood, as well as a deterioration in her quality of life and functionality. The patient denied any falls, fever, fluctuations in weight, or other significant symptoms.

At evaluation, the patient was partially disoriented, inattentive, with mild ataxia and dysarthria upon neurological examination. Her vital signs and the rest of the physical examination were without relevant findings. An extensive study of potential causes for delirium was conducted. A mild urinary tract infection was found and treated with amikacin. Hepatic encephalopathy, renal failure, intoxication, drug or medication withdrawal, and urinary or fecal retention were ruled out. Considering the history of mesothelioma, and the recent initiation of immunotherapy, a comprehensive neurological study was performed: Contrast-enhanced brain MRI revealed non-confluent supratentorial white matter microangiopathy, without evidence of vascular lesions or mesothelioma dissemination; electroencephalogram was normal; lumbar puncture showed no signs of encephalitis or leptomeningeal spread.

Further investigations revealed a suppressed thyroid-stimulating hormone (TSH), prompting a comprehensive evaluation of the thyroid axis. The results demonstrated elevated levels of peripheral thyroid hormones (total T4 and T3), absence of anti-thyroid-stimulating hormone receptor antibodies (TRAb) and anti-thyroid peroxidase (TPO), along with the presence of anti-thyroglobulin (Anti-Tg) antibodies. No additional endocrine irregularities were detected, and subsequent thyroid iodine uptake studies indicated low uptake. Ultimately, it was concluded that the etiology of the delirium was a thyrotoxicosis episode that developed in the context of autoimmune thyroiditis, secondary to ICI treatment.

**Figure 1:**
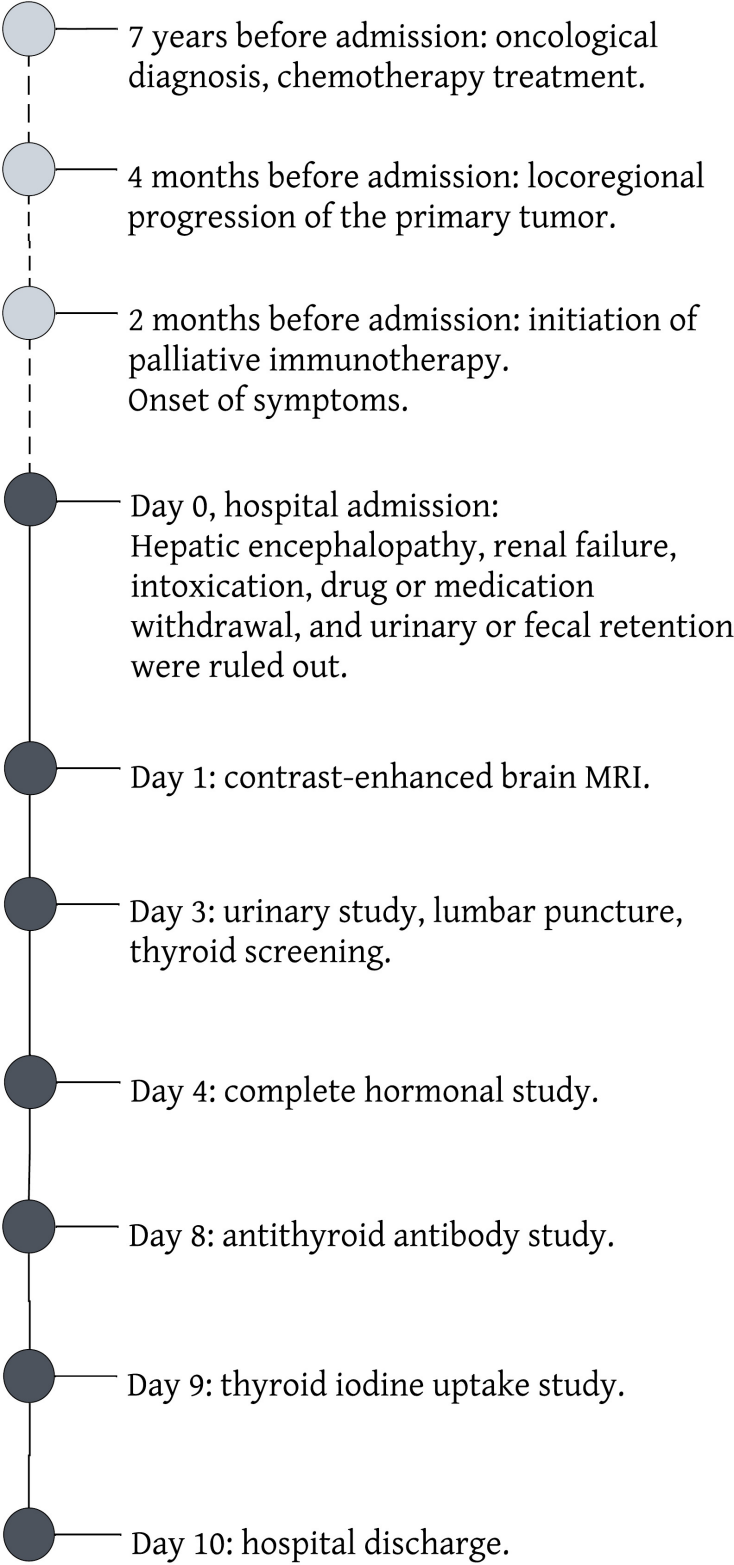
Timeline of the clinical case presented.

Symptomatic treatment with beta-blockers was initiated, leading to a gradual amelioration of neurological manifestations. Upon discharge, the patient was scheduled for outpatient monitoring by an endocrinologist and advised to continue her oncological regimen without modifications. **Figure 1** describes the clinical course of our patient.

## 3 Discussion

Qualitative impairment of consciousness (i.e., delirium) and neuropsychiatric symptoms can be manifestations of ICI adverse reactions (4–6). Additionally, patients can have multiple causes of impaired consciousness (7). Currently, there is a lack of consensus regarding a standardized approach to managing impaired consciousness in individuals undergoing therapy involving ICIs.

**Figure 2:**
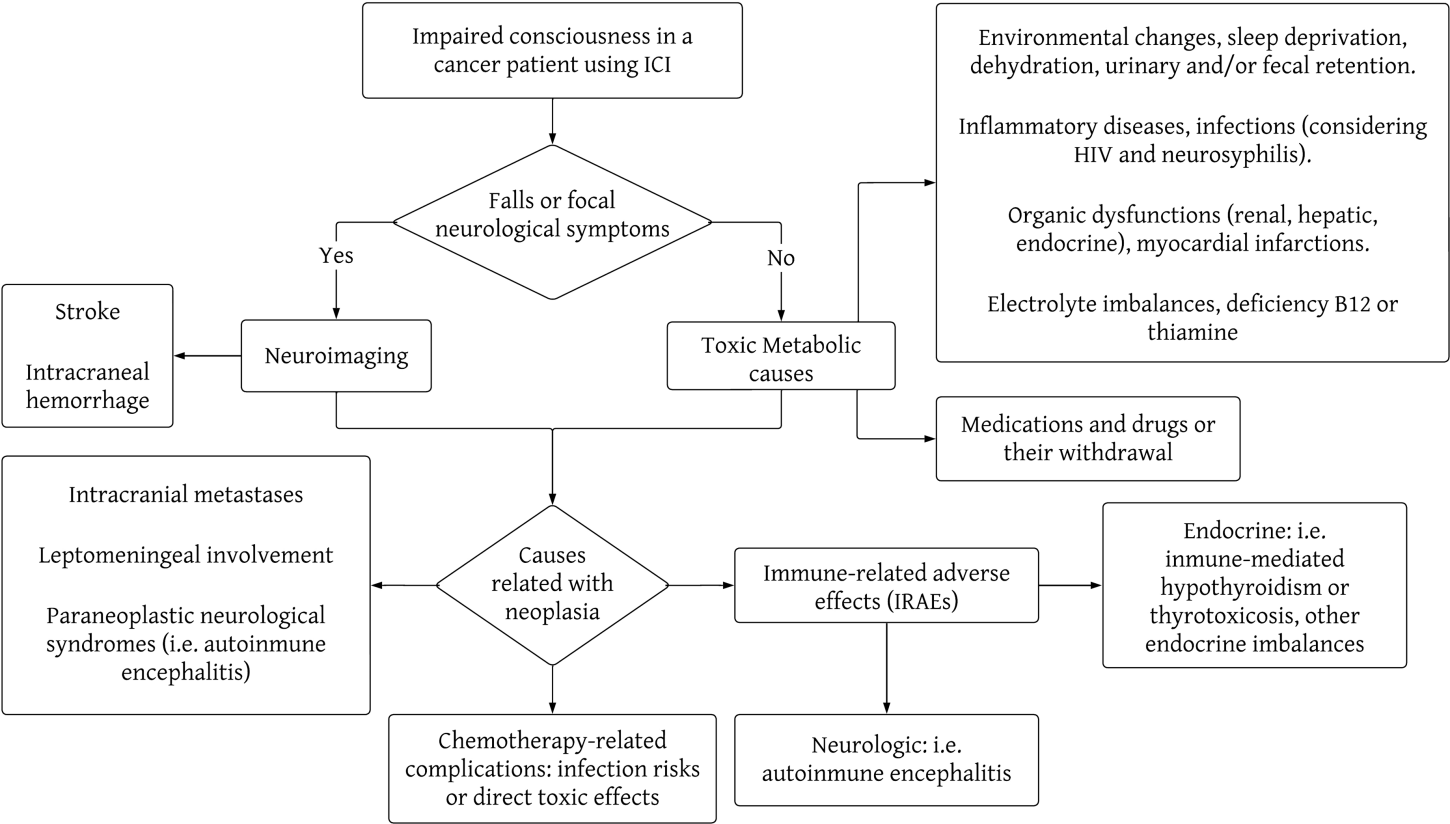
Flowchart of the most important causes of impaired consciousness in cancer patients undergoing therapy with immune checkpoint inhibitors. This diagram is not intended as a comprehensive list of all the causes of impaired consciousness, but a schematic approach to the diagnosis reasoning behind the study of these clinical causes.

**Figure 3:**
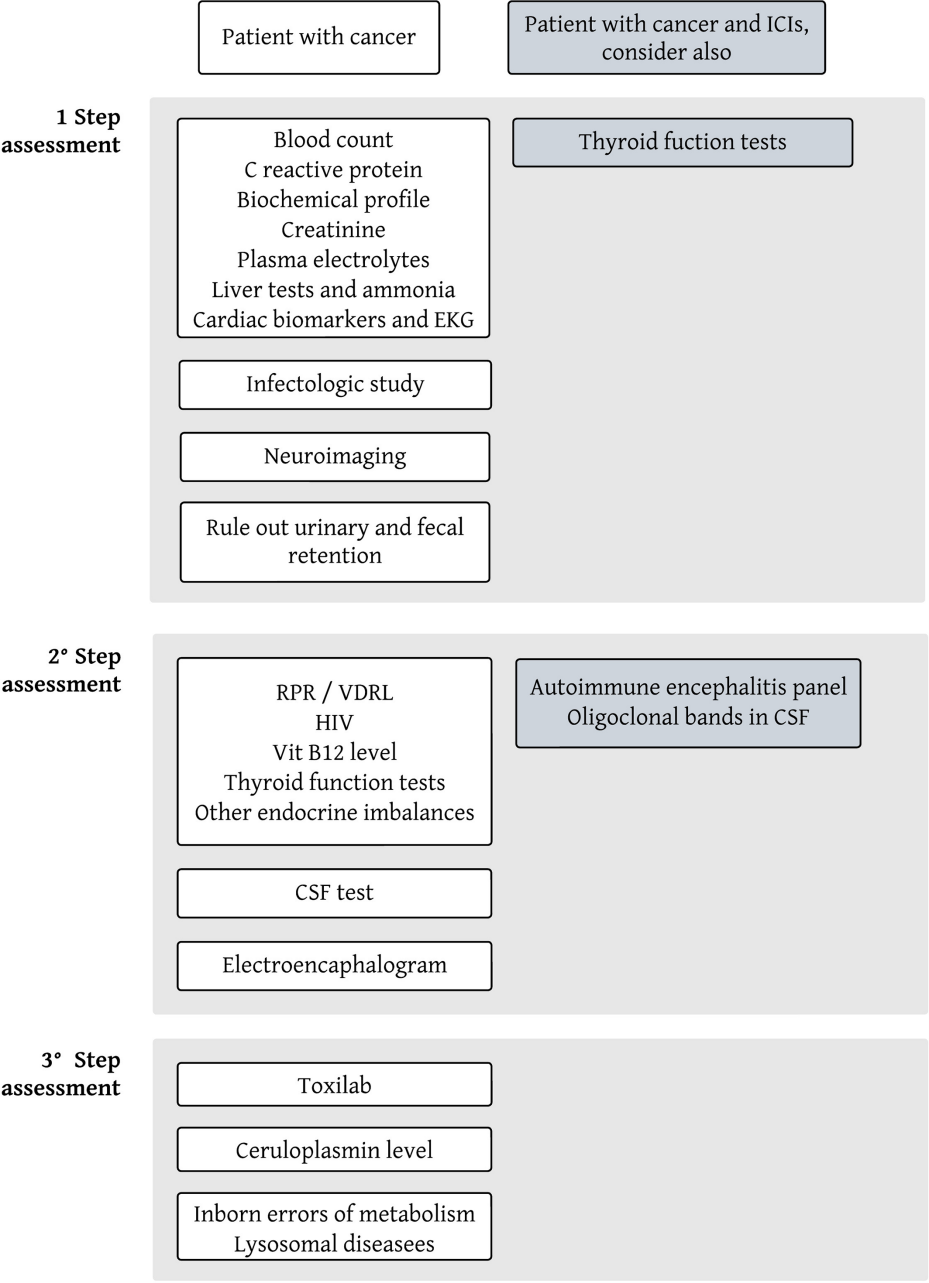
Proposed multistep approach for the laboratory and imaging workup of cancer patients using immune checkpoint inhibitors that consult because of impaired consciousness. This diagram should not be taken as rigid, and the approach should be modified and adapted to the clinical case being faced, according to the pre-test probability of each entity.

Delirium can be a difficult syndrome to diagnose in the presence of neuropsychiatric symptoms, such as emotional lability, delusions, and hallucinations, but it always presents with the particular characteristic of being fluctuating over time (3). Among the possible precipitating causes of delirium are infections, myocardial infarctions, inflammatory diseases (e.g., acute pancreatitis), neurological diseases (e.g. strokes), surgeries, organic dysfunctions (renal, hepatic, endocrine, hematological such as anemia, etc.), pain, sleep deprivation, dehydration, and electrolyte imbalances, medications and drugs or their withdrawal, urinary and/or fecal retention, and environmental changes (2). Neuropsychiatric symptoms and psychoses can be manifestations of medical conditions that should be actively ruled out, including thyroid diseases, vitamin B12 or thiamine deficiency (B1, Wernicke-Korsakoff Encephalopathy), Wilson’s Disease, neurosyphilis, HIV infection, brain tumors, some epilepsies, adverse effects of medications (such as systemic corticosteroids), and even rare diseases, such as lysosomal diseases (8, 9). In managing patients on immune checkpoint inhibitors (ICIs), clinicians must also explore potential complications directly linked to cancer, including neurological disease progression, intracranial metastases, leptomeningeal involvement, or the emergence of paraneoplastic neurological syndromes impacting the central nervous system (such as encephalitis, encephalomyelitis). Additionally, they should consider chemotherapy-related complications, like heightened infection risks or direct toxic effects. ICIs increase the likelihood of IRAEs, whether they be neurological (e.g., autoimmune encephalitis) or from other systems, where the symptoms relate to the central nervous system, such as endocrinopathies, renal failure, liver failure, etc. (4, 7, 10, 11). In **Figures 2**, **3** we propose a sequential approach to the oncologic patient that consults because of impaired consciousness, considering the list of causes previously described.

In our patient, although the initial study indicated a possible low urinary tract infection, this did not justify the diversity of neuropsychiatric symptoms associated with palpitations or heat intolerance, nor a temporal correlation with the initiation of ICI therapy. This finding prompted us to explore other possible causes following the list of etiologies previously described, seeking an alternative diagnosis that could unify the presentation of impaired consciousness in a patient with advanced cancer under ICI treatment. Therefore, we ruled out neurological causes, such as autoimmune encephalitis, intracerebral lesions, and leptomeningeal involvement secondary to oncological pathology, and studied endocrinological causes, confirming thyrotoxicosis.

The involvement of the thyroid in ICI-related IRAEs is very common, observed as asymptomatic laboratory abnormalities, primary immune-mediated hypothyroidism (13-16% in users of combined immunotherapy), thyrotoxicosis due to autoimmune glandular destruction (up to 8 to 10% of patients using combined immunotherapy) or Basedow-Graves disease. In general, these develop during the first months of treatment (1, 12, 13). Thyrotoxicosis refers to the clinical scenario that arises from an excess of thyroid hormones, associated with a wide variety of symptoms (14, 15). Among the most commonly reported neurological symptoms are mood alterations, such as irritability, anxiety, and depression, while psychotic symptoms have been very rarely reported: there is a series of 18 cases presented by the group led by B. Brownlie in 2000, and some isolated cases reports (16–21). Of these, stand out a case of thyrotoxicosis with hyperactive delirium and another one with hypomania and paranoia; we also noticed that the majority of cases reported with psychotic symptoms are of Graves disease in patients with preexistent psychiatric diseases, leading us to the conclusion that psychoses associated to thyrotoxicosis not caused by Graves disease is rare. With the previous evidence the incidence of psychoses in thyrotoxicosis has been estimated as approximately 1% (16). In our patient, thyrotoxicosis was secondary to thyroiditis associated with ICI use, which is consistent with the lower iodine uptake and the profile of autoantibodies found, a situation that we find unique with the actual available evidence.

Endocrine IRAEs can occur in 12 to 34% of patients, potentially affecting any gland in the body (with the thyroid being frequently affected), but their lethality is relatively low. This contrasts with neurological IRAEs, such as autoimmune encephalitis, which have an incidence of less than 3%, but a lethality of 3 to 16% (1, 22, 23). The oncological prognosis, beyond direct mortality from the complication itself, is also diametrically different between these two groups: recent evidence shows that the occurrence of thyroid IRAEs is associated with better treatment response, and even longer survival, compared to patients who do not have them (24–26); on the other hand, the occurrence of neurological IRAEs requires ICI discontinuation, and the initiation of treatment involving potent immunosuppression with systemic corticosteroids and intravenous immunoglobulins, among other options, that can further worsen oncologic prognosis (1, 22, 27). In the case of thyroiditis, treatment consists of symptomatic management with beta-blockers, adding prednisolone only if there is associated neck pain, and temporarily suspending ICI use solely if the symptoms are poorly tolerated, without being an indication for discontinuing therapy (12, 27).

Unfortunately, follow-up was lost after discharge and we do not have information about further symptom resolution and TSH level monitoring after immune checkpoint inhibitor therapy was resumed. Although this poses a limitation for our case report, we were able to present our approach to oncologic patients being treated with ICIs that present with impaired consciousness, such as the one presented.

## 4 Conclusion

This case highlights the intricate diagnostic challenge of impaired consciousness in cancer patients who use ICIs, especially considering the wide spectrum of possible etiologies. In this context, the recognition of delirium symptoms in relation to thyrotoxicosis as an IRAE serves as a pertinent example, demonstrating the differential impact of endocrine IRAEs versus neurological ones on patient outcomes and oncological prognosis. An organized and multisystemic diagnostic approach is crucial to reach the correct diagnosis, and thus, implement appropriate management for both the acute pathology, and the underlying oncological disease.

## Data Availability

The original contributions presented in the study are included in the article/supplementary material. Further inquiries can be directed to the corresponding authors.
